# Sarcopenia is not associated with hypertension, but sarcopenic obesity increases risk of hypertension: a 7-year cohort study

**DOI:** 10.3389/fpubh.2024.1479169

**Published:** 2025-01-15

**Authors:** Runfen Du, Junchao Yuan, Yunda Huang, Guihua Jiang, Zhiping Duan, Hong Yang, Wei Huang

**Affiliations:** ^1^Department of Geriatrics, The Third People’s Hospital of Yunnan Province, Kunming, Yunnan, China; ^2^Department of Endocrinology, The First People’s Hospital of Yunnan Province, Kunming, Yunnan, China

**Keywords:** obesity, sarcopenia, sarcopenic obesity, hypertension, CHARLS

## Abstract

**Background:**

Sarcopenia, sarcopenic obesity, and hypertension are all widespread public health problems in middle-aged and older populations, and their association is controversial. The purpose of this study is to analyze the relationship between obesity, sarcopenia, and sarcopenic obesity with hypertension in a middle-aged and older community population in China through a large-scale longitudinal design.

**Methods:**

In this cohort study with 7 years of follow-up, the study population was drawn from participants in the China Health and Retirement Longitudinal Study (CHARLS) in 2011 and followed up in 2013, 2015, and 2018. The diagnostic criteria for sarcopenia were based on the consensus recommendations issued by the Asian Working Group for Sarcopenia (AWGS) in 2019. The diagnosis of obesity is based on body mass index and waist circumference. Sarcopenic obesity is defined as the coexistence of sarcopenia and obesity. Cox proportional risk regression models were used to analyze the association of obesity, sarcopenia, and sarcopenic obesity with hypertension.

**Results:**

A total of 7,301 participants with a mean age of 58 ± 8.8 were enrolled in the study, and 51.9% females. A total of 1,957 participants had a new onset of hypertension after 7 years of follow-up. In a multifactorial analysis, obesity and sarcopenic obesity were associated with hypertension; hazard ratios (HRs) and 95% confidence intervals (CIs) were 1.67 (1.43 ~ 1.96), *p* < 0.001, and 1.61 (1.09 ~ 2.37), *p* = 0.017. Sarcopenia and hypertension were not significantly associated; the HR and 95% CI were 1.17 (0.9 ~ 1.52), *p* = 0.23.

**Conclusion:**

There is no significant correlation between sarcopenia and hypertension, but obesity and sarcopenic obesity increase the risk of hypertension. Targeted management of middle-aged and older people with sarcopenic obesity is needed in public health efforts.

## 1 Introduction

Hypertension is a common chronic disease in the older population, and according to the Global Burden of Hypertension Disease Report (1), it is projected that the global burden of disease from hypertension may exceed 1.6 billion people by 2025. Hypertension increases the risk of diseases such as cardiovascular disease, stroke, kidney disease, and retinopathy, reduces quality of life, and significantly increases the risk of death (2–4). Hence, delving deeper into the risk factors for hypertension becomes crucial for early detection and intervention.

Sarcopenia is a chronic condition associated with aging that is characterized by a reduction in skeletal muscle mass with a concomitant decline in muscle strength or somatic function and is associated with adverse events such as falls and fractures, increasing disability and mortality rates, and seriously affecting the quality of life of older people (5). A large longitudinal cohort study from China Health and Retirement Longitudinal Study (CHARLS) found that higher grip strength was an independent protective factor for hypertension in a middle-aged and older population (6). Sarcopenic obesity is the presence of sarcopenia and obesity in the same individual, with the two acting synergistically to affect health and lead to serious adverse outcomes (7–10). It has been established that obesity significantly increases the risk of hypertension through metabolic disorders, insulin resistance, and atherosclerosis (11). In addition, in obese patients, fat stored in the viscera, in particular, releases pro-inflammatory cytokines that cause the body to be in a state of chronic inflammation, and the aging process is associated with chronic low-grade inflammation called inflammatory aging (12). Inflammatory aging plays a prominent role in age-related chronic diseases such as atherosclerosis, insulin resistance, and sarcopenia, while this low-grade inflammation is one of the key factors contributing to the reduction of skeletal muscle mass (13). Numerous animal studies have provided compelling evidence for a causal role of inflammation in the pathogenesis of hypertension (14). Inflammation promotes intrarenal angiotensin II production by infiltrating inflammatory cells and renal tubular epithelial cells, which in turn further causes hypertension (15). There is some mechanistic overlap between sarcopenia, obesity, and sarcopenic obesity and hypertension.

A large number of studies have confirmed that obesity is a risk factor for hypertension (16–18), but there are discrepancies in the association between sarcopenia and sarcopenic obesity with hypertension. Myasthenia gravis obesity was similarly found to be a risk factor for hypertension in a US cohort study (19) that included 1,019 participants. However, a study by Dos Santos et al. (20) found that sarcopenia and sarcopenic obesity were not associated with arterial blood pressure. In an Iranian cross-sectional study (21), it was shown that obesity was associated with hypertension, whereas sarcopenia and sarcopenic obesity were not significantly associated with hypertension. Different studies may have inconsistent results due to differences in race, geography, lifestyle, and dietary habits. China accounts for more than 1/6 of the world’s population, and it is unclear whether sarcopenia and sarcopenic obesity are risk factors for hypertension in Chinese community populations. For this reason, we conducted a large 7-year CHARLS-based cohort study aimed at investigating the longitudinal association between sarcopenia and sarcopenic obesity and new-onset hypertension in a middle-aged and older Chinese community population, to provide a new evidence for the management of hypertension risk in such populations.

## 2 Methods

### 2.1 Study population

**Figure 1:**
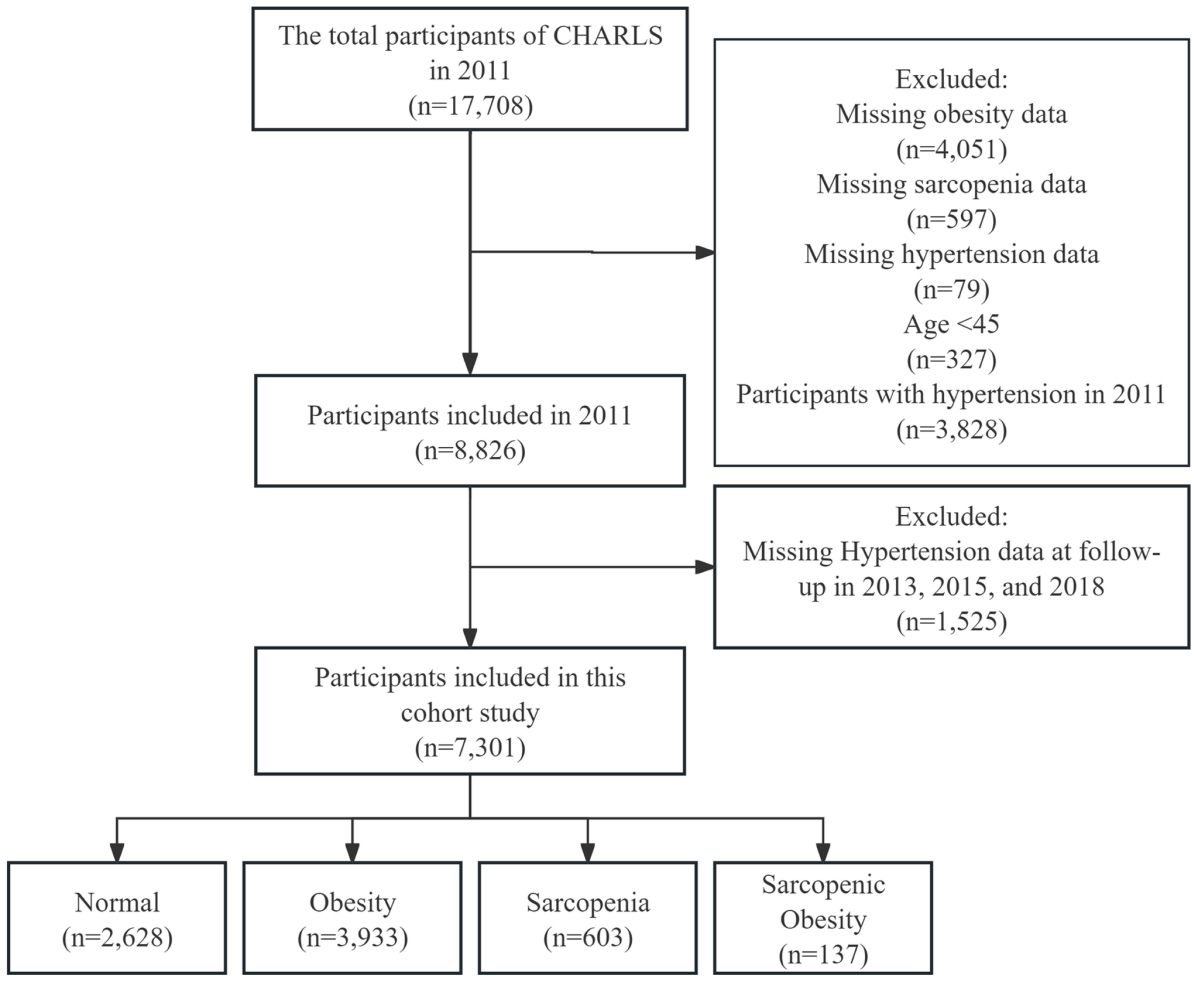
Flow chart of the screening of participants.

Participants in this cohort study were drawn from the China Health and Retirement Longitudinal Study (CHARLS) 2011, 2013, 2015 and 2018 survey, which collects a high-quality microdata set representative of middle-aged and older Chinese adults aged 45 years and older at the household and individual level to analyze the aging of the Chinese population (22). We developed a few exclusion criteria: (1) missing data on obesity (4,051), sarcopenia (597), and hypertension (79) at baseline; (2) age < 45 years (327); (3) with hypertension at baseline (3,828); and (4) missing data on hypertension at follow-up in 2013, 2015, and 2018 (1,525). Finally, 7,301 participants were included in this cohort study (Figure 1).

The CHARLS study was approved by the Peking University Biomedical Ethics Committee (approval number: IRB00001052-11015), and each participant signed an informed consent form (22). The study was conducted in accordance with the Strengthening the Reporting of Observational Studies in Epidemiology (STROBE) recommendations for cohort studies (23).

### 2.2 Assessment of obesity, sarcopenia, and sarcopenic obesity

Obesity status was assessed using body mass index (BMI) and waist circumference; specifically, obesity was defined when BMI was ≥28 kg/m^2^ (24) or when waist circumference was ≥85 cm for men and ≥ 80 cm for women (25). The participants’ height and weight were measured in the standing position using Seca™ 213 height meters (China Seca Hangzhou Co., Ltd.) and Omron™ HN-286 weight scales (Kerui Technology Yangzhou Co., Ltd.), measuring waist circumference Using a Soft Ruler.

The assessment of sarcopenia status was based on the consensus recommendations issued by the Asian Working Group for Sarcopenia (AWGS) in 2019 (26), which are more appropriate for the Chinese population. Specifically, sarcopenia was defined as a decrease in skeletal muscle mass combined with a decrease in physical performance or a decrease in muscle strength. As there were no data on DXA or BIA in CHARLS, we employed the muscle mass equations that have been demonstrated to exhibit satisfactory concordance with DXA in the Chinese population to calculate the appendicular skeletal muscle mass (ASM) of the participants (27):


$$\begin{array}{c} ASM=0.193\times \mathrm{weight}\left(\mathrm{kg}\right)+0.107\times \mathrm{height}\left(\mathrm{cm}\right)\\ -4.157\times \mathrm{sex}-0.037\times \mathrm{age}\left(\mathrm{year}\right)\\ -2.631\;\left[\mathrm{Sex}\;\mathrm{for male is}\;1\mathrm{,for female is}\;2\right]\text{.} \end{array}$$

The skeletal muscle mass index (SMI) was calculated as follows: SMI = ASM / height^2^. The cut-off value for low SMI was defined as the lowest 20% of SMI by sex in the study population: 7 kg/m^2^ for male and 5.28 kg/m^2^ for female. Muscle strength was assessed using grip strength by using the Yuejian™ WL-1000 dynamometer (Nantong Yuejian Physical Testing Equipment Co., Ltd.), and participants took turns measuring grip strength twice for each hand. As recommended by the AWGS 2019, low muscle strength was defined as a maximum grip strength value of <28 kg in males or < 18 kg in females in four measurements. Physical function was assessed by 5 chair stand tests and 5 m gait speed, with low physical function defined as 5 chair stand tests ≥12 s or gait speed <1.0 m/s. The diagnostic criteria for sarcopenic obesity are based on an the most recent expert consensus published in 2022 by the European Society for Clinical Nutrition and Metabolism (ESPEN) and the European Association for the Study of Obesity (EASO): sarcopenic obesity is defined as the coexistence of obesity and sarcopenia (28, 29).

### 2.3 Assessment of hypertension

Hypertension was assessed based on the question in the questionnaire, “Have you been diagnosed with hypertension by a doctor?” and the time of the onset of hypertension at the follow-up was derived from the question, “When was the condition first diagnosed or known by yourself?” Hypertension was considered to be present when answering yes to the above question or when the time variable was not missing. For missing time to diagnosis of hypertension (*n* = 73), incident time was defined as the mean time to incident hypertension in other participants during the follow-up period.

### 2.4 Covariates

The covariates included in our cohort study were demographic characteristics, lifestyle, chronic diseases, and other blood test indicators. Demographic characteristics included gender, age, education, marital status, and place of residence. Lifestyle included smoking and drinking. Chronic disease data came from the questionnaire, “Have you been diagnosed with a chronic disease by a doctor?” The results of the questionnaire include dyslipidemia, diabetes mellitus, and kidney disease. Other blood test data included estimated glomerular filtration rate (eGFR), total triglycerides (TG), high-density lipoprotein cholesterol (HDL-c), and low-density lipoprotein cholesterol (LDL-c).

### 2.5 Statistical analysis

All participants were divided into four groups: normal, obesity, sarcopenia, and sarcopenic obesity. Continuous variables were expressed as the mean ± standard deviation (SD) or median and interquartile range (IQR), and categorical variables were expressed as counts and percentages. The t-tests, chi-square tests, ANOVA, or non-parametric tests were used to analyze the baseline characteristics of participants. Univariate and multivariate Cox proportional hazards regression models were used to estimate hazard ratios (HRs) and 95% confidence intervals (CIs) for hypertension in different groups. Covariates included in the multifactor Cox proportional hazards regression model include: gender, age, education, marital status, residence, smoking, drinking, dyslipidemia, diabetes mellitus, kidney disease, eGFR, TG, HDL-c, and LDL-c. Cumulative risk curves for the development of hypertension were plotted using Kaplan–Meier curves, and differences between groups were analyzed using log-rank tests. Finally, considering that age, smoking, and renal function status are all strongly associated with hypertension and that gender may also be a potential influence (30), we performed subgroup analyses of participants with different gender, age, smoking, and eGFR status to explore interactions.

All the analyses were performed with the statistical software Stata/MP version 17.0 (StataCorp LP, College Station, TX, USA) and Free Statistics software versions 1.9.2. The level of statistical significance was set at *p* < 0.05 (two-sided).

## 3 Results

### 3.1 Baseline characteristics of study participants

This cohort study ultimately included 7,301 participants, with a mean age of 58 years (SD 8.8) and 51.9% females. Table 1 shows the baseline characteristics of all participants. At baseline, 3,933 participants were diagnosed with obesity, 603 with sarcopenia, and 137 with sarcopenic obesity. Overall, obese participants had the highest prevalence of diabetes and dyslipidemia and had higher TG and lower HDL-c, while sarcopenia participants had the highest HDL-c levels, all *p* < 0.001. 84.7% of the sarcopenic obesity group were female, and the mean age was 70.5 ± 9.1, which was significantly higher than the mean age of the other groups, p < 0.001. In addition, participants in the sarcopenic obesity group were less likely to be married, less likely to smoke, had the least amount of diabetes and kidney disease, and had the lowest LDL-c levels, all *p* < 0.05.

**Table 1:** Baseline characteristics of participants in this study according to obesity, sarcopenia, and sarcopenic obesity.

	Total (*n* = 7,301)	Normal (*n* = 2,628)	Obesity (*n* = 3,933)	Sarcopenia (*n* = 603)	Sarcopenic Obesity (*n* = 137)	*p*-value
Gender, *n* (%)						<0.001
Female	3,792 (51.9)	881 (33.5)	2,466 (62.7)	329 (54.6)	116 (84.7)	
Male	3,509 (48.1)	1,747 (66.5)	1,467 (37.3)	274 (45.4)	21 (15.3)	
Age, year	58.0 ± 8.8	57.1 ± 8.1	56.9 ± 8.3	66.4 ± 8.5	70.5 ± 9.1	<0.001
Education, *n* (%)						<0.001
Primary school or below	4,948 (67.8)	1,740 (66.2)	2,556 (65)	528 (87.6)	124 (90.5)	
Middle school	1,566 (21.4)	588 (22.4)	914 (23.2)	54 (9)	10 (7.3)	
High school or above	787 (10.8)	300 (11.4)	463 (11.8)	21 (3.5)	3 (2.2)	
Marital, *n* (%)						<0.001
Other	709 (9.7)	209 (8)	320 (8.1)	125 (20.7)	55 (40.1)	
Married	6,592 (90.3)	2,419 (92)	3,613 (91.9)	478 (79.3)	82 (59.9)	
Residence, *n* (%)						<0.001
Urban areas	1,154 (15.8)	317 (12.1)	772 (19.6)	51 (8.5)	14 (10.2)	
Rural areas	6,140 (84.2)	2,307 (87.9)	3,159 (80.4)	551 (91.5)	123 (89.8)	
Smoke, *n* (%)	2,898 (39.7)	1,416 (53.9)	1,216 (30.9)	241 (40)	25 (18.4)	<0.001
Drink, *n* (%)						<0.001
Never or rarely	4,468 (82.5)	1,373 (77.9)	2,598 (84.8)	388 (83.3)	109 (89.3)	
Less than once a month	586 (10.8)	235 (13.3)	302 (9.9)	42 (9)	7 (5.7)	
More than once a month	361 (6.7)	154 (8.7)	165 (5.4)	36 (7.7)	6 (4.9)	
Dyslipidemia, *n* (%)	369 (5.1)	82 (3.2)	280 (7.2)	4 (0.7)	3 (2.2)	<0.001
Diabetes, *n* (%)	235 (3.2)	53 (2)	167 (4.3)	15 (2.5)	0 (0)	<0.001
Kidney disease, *n* (%)	421 (5.8)	177 (6.8)	208 (5.3)	32 (5.4)	4 (2.9)	0.037
eGFR, mL/(min × 1.73 m^2^)	110.6 ± 29.0	110.2 ± 27.6	111.3 ± 30.1	107.6 ± 27.4	109.3 ± 30.5	0.074
TG, mg/dL	100.9 (72.6, 146.9)	86.7 (63.7, 124.1)	115.9 (82.3, 169.9)	83.6 (63.1, 112.2)	103.1 (77.2, 137.4)	<0.001
HDL-c, mg/dL	52.0 ± 15.3	55.5 ± 15.6	48.5 ± 13.9	59.5 ± 16.7	55.7 ± 14.6	<0.001
LDL-c, mg/dL	115.9 ± 34.0	112.5 ± 32.5	118.5 ± 35.1	112.1 ± 31.8	120.8 ± 34.2	<0.001
Height, cm	158.2 ± 8.4	159.5 ± 7.7	158.4 ± 8.3	154.1 ± 9.0	148.9 ± 10.2	<0.001
Weight, kg	58.4 ± 10.7	54.5 ± 6.5	63.6 ± 10.3	44.5 ± 5.7	45.1 ± 5.3	<0.001
BMI, kg/m^2^	23.3 ± 3.6	21.4 ± 1.8	25.3 ± 3.3	18.7 ± 1.5	20.4 ± 1.8	<0.001
BMI ≥ 28 kg/m^2^	600 (8.3)	0 (0)	600 (15.4)	0 (0)	0 (0)	<0.001
Waist circumference, cm	83.4 ± 11.8	75.4 ± 10.5	90.4 ± 7.8	72.3 ± 7.8	85.7 ± 4.4	<0.001
SMI, kg/m^2^	6.7 ± 1.1	6.7 ± 0.9	7.0 ± 1.1	5.6 ± 1.0	5.2 ± 0.7	<0.001
Gait speed, m/s	1.4 ± 0.5	1.5 ± 0.4	1.4 ± 0.5	1.2 ± 0.5	1.1 ± 0.4	<0.001
5-Time chair stand test, s	10.4 ± 4.0	9.6 ± 3.1	10.4 ± 3.8	13.5 ± 5.4	15.0 ± 5.3	<0.001
Handgrip strength, kg	33.3 ± 10.3	35.6 ± 9.3	33.4 ± 10.3	25.6 ± 8.5	21.8 ± 8.1	<0.001

eGFR, estimated glomerular filtration rate; TG, Total triglycerides; HDL-c, high-density lipoprotein cholesterol; LDL-c, low-density lipoprotein cholesterol; BMI, body mass index; ASM, appendicular skeletal muscle mass.

### 3.2 Association between obesity, sarcopenia, and sarcopenic obesity with hypertension

**Figure 2:**
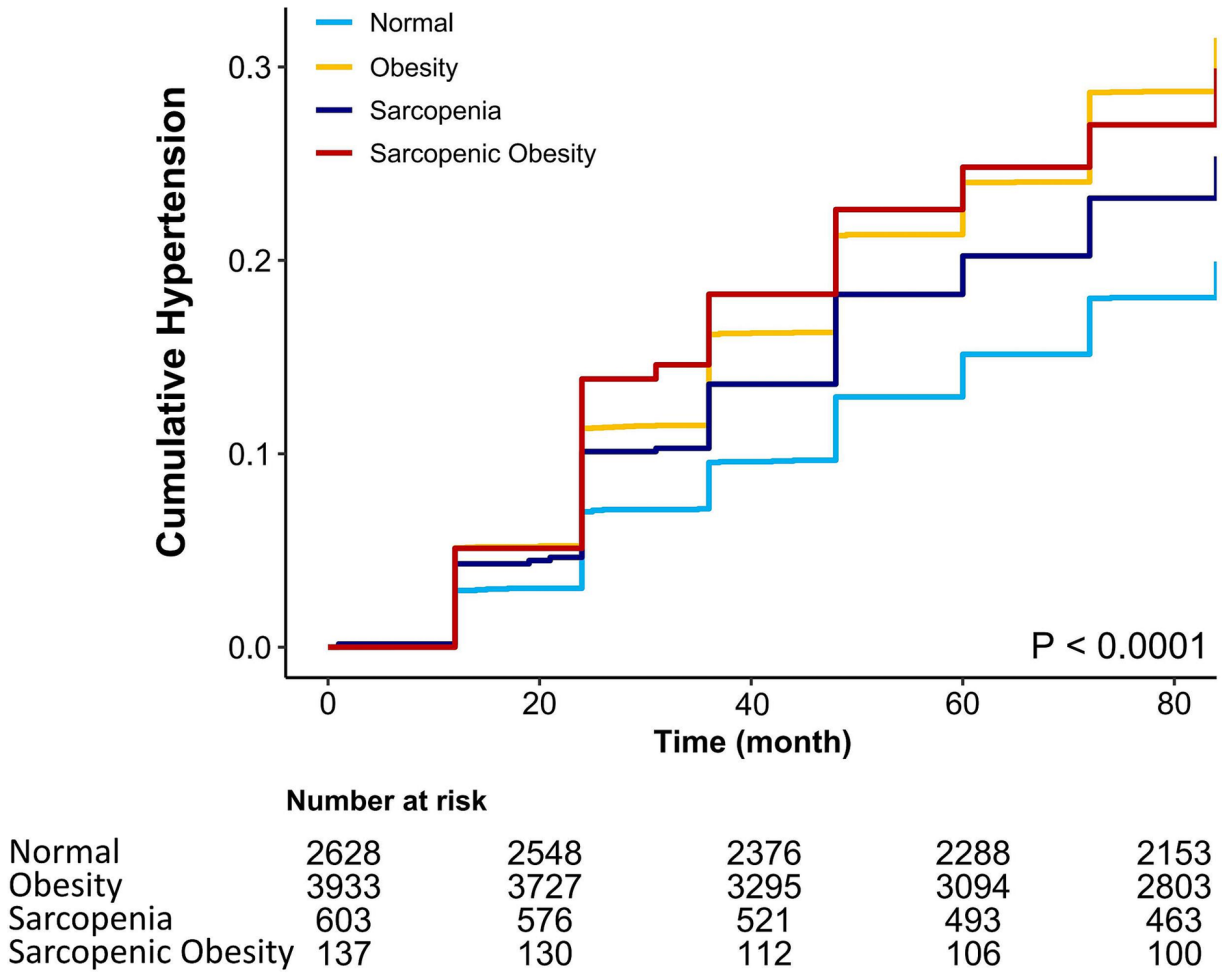
The cumulative risk of hypertension increases over time (log-rank test, *p* < 0.0001). Light blue indicates normal population, yellow indicates obesity, dark blue indicates sarcopenia, and red indicates sarcopenic obesity.

After following up for 7 years, there were 1,957 cases of hypertension, corresponding to 26.8% of all participants. Table 2 show the prevalence, HR, and 95%CI of hypertension in obesity, sarcopenia, and sarcopenic obesity participants, using normal participants as references. The prevalence of hypertension in normal, obesity, sarcopenia, and sarcopenic obesity participants were 19.9, 31.5, 25.4, and 29.9%. In univariate analyses, the HRs and 95% CIs for hypertension in participants with obesity, sarcopenia, and sarcopenic obesity were 1.7 (1.54 ~ 1.88), 1.33 (1.11 ~ 1.59), and 1.63 (1.19 ~ 2.24), respectively, all *p* < 0.01. In multivariate analysis after adjustment for all covariates, obesity and sarcopenic obesity were associated with hypertension; HRs and 95% CIs were 1.67 (1.43 ~ 1.96) and 1.61 (1.09 ~ 2.37), both *p* < 0.05. Sarcopenia and hypertension were not significantly associated, HR and 95% CI was 1.17 (0.9 ~ 1.52), *p* = 0.23. Kaplan–Meier curves showed that the cumulative risk of hypertension increased over time; Participants with obesity and sarcopenic obesity possessed a higher cumulative risk of hypertension (log-rank test, *p* < 0.0001) (Figure 2).

**Table 2:** Univariate and multivariate analysis the relationship between obesity, sarcopenia and sarcopenic obesity with hypertension.

	n event (%)	Univariate		Multivariate	
		HR (95%CI)	*p*-value	HR (95%CI)	*p*-value
Normal	524 (19.9)	1 (Ref)		1 (Ref)	
Obesity	1,239 (31.5)	1.7 (1.54 ~ 1.88)	<0.001	1.67 (1.43 ~ 1.96)	<0.001
Sarcopenia	153 (25.4)	1.33 (1.11 ~ 1.59)	0.002	1.17 (0.9 ~ 1.52)	0.23
Sarcopenic Obesity	41 (29.9)	1.63 (1.19 ~ 2.24)	0.003	1.61 (1.09 ~ 2.37)	0.017

Multivariate: adjusted for gender, age, education, marital, residence, smoke, drink, dyslipidemia, diabetes, kidney disease, eGFR, TG, HDL-c, LDL-c.

HR, hazards ratio; CI, confidence interval; eGFR, estimated glomerular filtration rate; TG, Total triglycerides; HDL-c, high-density lipoprotein cholesterol; LDL-c, low-density lipoprotein cholesterol.

**Figure 3:**
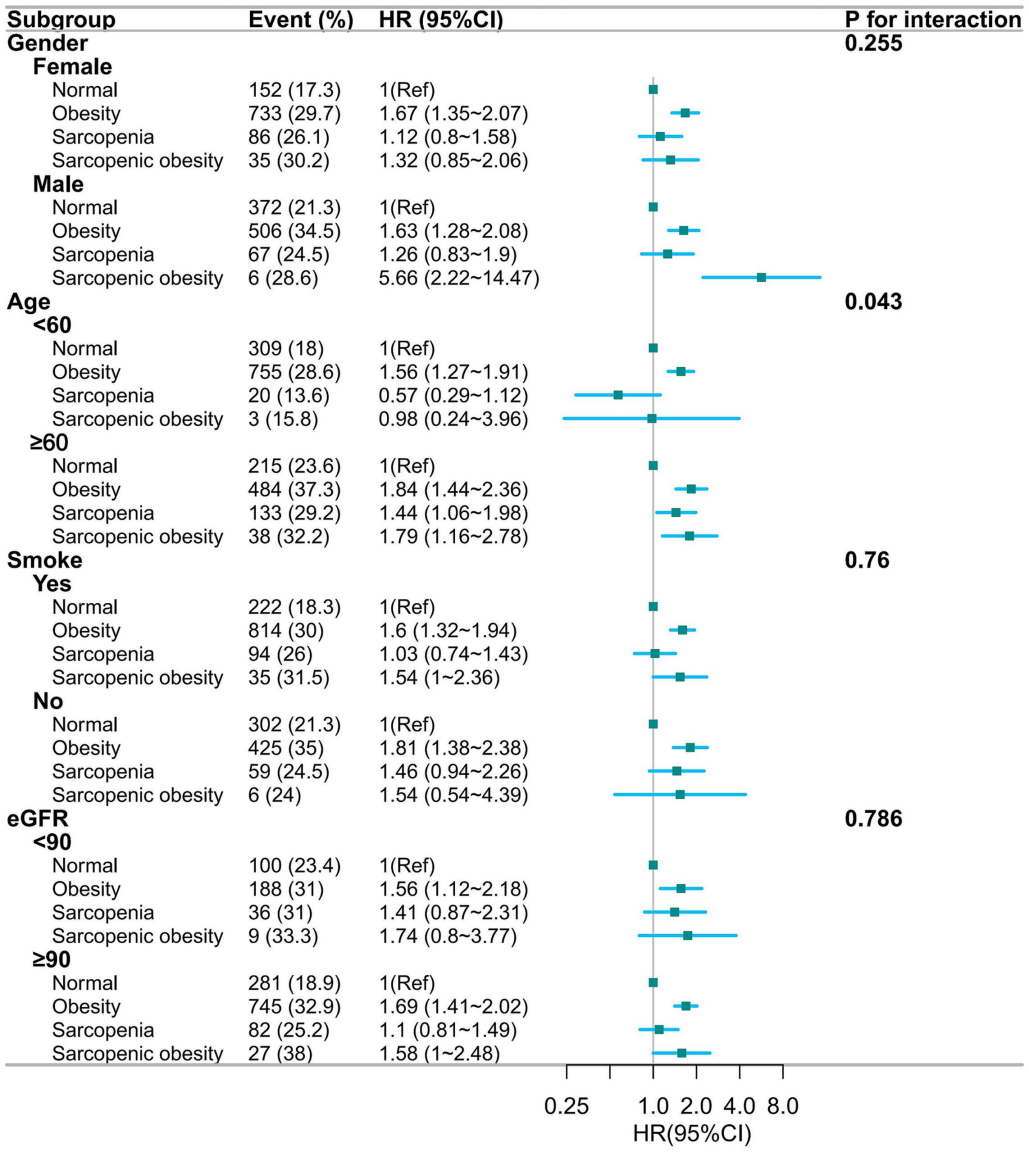
Forest plot of multifactorial subgroup analyses based on gender, age, smoking, and eGFR grouping, adjusted for gender, age, education, marital, residence, smoke, drink, dyslipidemia, diabetes, kidney disease, eGFR, TG, HDL-c, LDL-c. HR, hazards ratio; CI, confidence interval; eGFR, estimated glomerular filtration rate; TG, Total triglycerides; HDL-c, high-density lipoprotein cholesterol; LDL-c, low-density lipoprotein cholesterol.

Figure 3 shows the results of the multifactorial subgroup analysis. The association between obesity, sarcopenia, and sarcopenic obesity with hypertension was similar among participants of different genders, smoking, and eGFR status. However, we found that the effects of sarcopenia and sarcopenic obesity on hypertension appeared to differ between those aged less than 60 years and those aged more than 60 years.

## 4 Discussion

In our cohort study, the diagnostic criteria for sarcopenia were based on the recommendations of AWGS 2019, and the diagnostic criteria for sarcopenic obesity were based on the expert consensus published jointly by ESPEN and EASO in 2022. We used the representative CHARLS database to analyze the associations between obesity, sarcopenia, and sarcopenic obesity with the risk of new-onset hypertension in a middle-aged and older Chinese community-based population. After adjusting for all covariates, obesity and sarcopenic obesity were significantly associated with hypertension, and there was no significant association between sarcopenia and hypertension.

In our study, sarcopenic obesity increased the risk of hypertension, and sarcopenia was not significantly associated with hypertension. A cross-sectional study by Dutra et al. (31) of women in a Brazilian community found that sarcopenic obesity was a risk factor for hypertension, and that the prevalence of sarcopenic obesity was higher than in other studies, but the number of subjects enrolled was relatively small, which makes it difficult to discuss the prevalence. A large cross-sectional study in northern China (32) included 14,926 adults aged 35 ~ 74 years and found a high prevalence of sarcopenic obesity (65.1%), which is associated with hypertension, diabetes mellitus, and lipid metabolic abnormalities. However, a cross-sectional survey by Pasdar et al. in Iranian Kurds (21) showed that sarcopenia and sarcopenic obesity were not associated with hypertension. Another cross-sectional study, also from northern China, including 1,082 participants (33), found that men of advanced age, physical inactivity, diabetes mellitus, and absence of hypertension may have a higher prevalence of sarcopenia, and this study had a smaller sample size and was limited to the local area of Bengbu. Another cross-sectional study in Korea (34) found that subjects with obese sarcopenia appeared to have a greater risk of hypertension than subjects with obesity or sarcopenia alone. So the association between sarcopenia, sarcopenic obesity, and hypertension may vary in different countries because sarcopenia may be influenced by economic level, medical level, and genetic factors.

Definitions of obesity and measurement techniques (BMI and waist circumference), while consistent with standard practice, have some limitations. For example, BMI does not distinguish between fat and muscle mass, which may affect its accuracy in identifying sarcopenic obesity. In our study population, the majority of both obesity and sarcopenic obesity were abdominal obesity, with only 15.4% of obesity participants having a BMI ≥28 kg/m^2^ and all sarcopenic obesity participants having a BMI <28 kg/m^2^. Similar to our findings, an epidemiological study on sarcopenic obesity based on the Western Cohort Population conducted in China (35) found that defining obesity by waist circumference found a prevalence of 7.22% for sarcopenic obesity, whereas the prevalence of sarcopenic obesity in obesity defined by BMI was only 0.63%. To our knowledge, in a retrospective cohort study in China that included 74,955 participants (36), abdominal obesity was found to be about two times more prevalent than hypertension in those with normal abdominal circumference, which is a risk factor for hypertension. Similarly, in a cohort study of risk factors for hypertension in the United States (37), which included 3,475 participants, centripetal obesity significantly increased the risk of developing hypertension.

Our study also found a significant gender difference in the prevalence of sarcopenic obesity, with a prevalence of 84.7% (116/137) in females. This gender difference may be related to age-related changes in sex hormone levels; the mean age of the sarcopenic obesity group was 70.5 ± 9.1 years, and most of the women were in menopause. In addition, women (62.7%) were more likely than men (37.3%) to be purely obese participants in our baseline profile, which may also account for the gender difference. It has been found that a decrease in estrogen levels associated with menopause is associated with a decrease in muscle mass and an increase in total body fat (38). During menopause, changes in hormone levels may lead to the release of pro-inflammatory cytokines, and this induced systemic mild inflammation can promote the loss of muscle mass. In men, testosterone can promote muscle regeneration by activating satellite cells, enhancing muscle protein synthesis, and increasing androgen receptor expression (39). Decreasing testosterone levels with increasing age may have a side effect on muscle mass and fat distribution in older adults (40).

The pathogenesis of obesity and hypertension is complex. Obesity and sarcopenic obesity have been found to be associated with the development of hypertension through the following common underlying mechanisms (e.g., chronic inflammation, insulin resistance). Both obesity and sarcopenic obesity trigger the release of inflammatory factors (41), which promotes muscle protein catabolism and accelerates the progression of sarcopenia. These inflammatory factors can also affect the sympathetic nervous system and the renin-angiotensin-aldosterone system, leading to elevated blood pressure. Low serum lipocalin levels in obese patients contribute to hypertension by increasing insulin resistance, and there is growing evidence that insulin resistance is associated with sarcopenia (42). Our study found that sarcopenia alone did not increase the risk of hypertension, but sarcopenic obesity increased the risk of hypertension, which is consistent with the findings of Dutra et al. (31). Our study implies a possible threshold effect when sarcopenia increases the risk of hypertension only when combined with obesity, while the risk of hypertension is not increased in sarcopenic populations without combined obesity. The specific thresholds and mechanisms of BMI or waist circumference that lead to an increased risk of hypertension in the sarcopenia population remain to be further investigated.

In our subgroup analyses, the effects of sarcopenia and sarcopenic obesity on hypertension differed significantly between those aged >60 and < 60 years. This may be related to the age characteristics of our study population. In our study, <60 years of age accounted for fewer participants with sarcopenia (147/603) and sarcopenic obesity (19/137). Sarcopenia and hypertension share a variety of potential pathogenic mechanisms (e.g., insulin resistance, decreased physical activity, chronic inflammation, inadequate protein intake). Chronic inflammation, particularly the production of catabolic cytokines (41), is a major mechanism leading to sarcopenia and age-related chronic diseases, including hypertension (34). The common pathway of reduced physical activity with age, leading to skeletal muscle mitochondrial autophagy hyperfunction and the accumulation of oxidative toxicants, which leads to muscle damage, may explain why hypertension is more prevalent in participants with sarcopenia or sarcopenic obesity over the age of 60 years, and the prevalence increases with age. However, the interaction of age with this needs further study.

To the best of our knowledge, this is the first representative cohort study with a large sample size based on a community-based population of middle-aged and older people in China. We found that sarcopenia is not associated with the risk of hypertension, but sarcopenic obesity increases the risk of hypertension. The findings of this study highlight the significant impact of sarcopenic obesity on the risk of hypertension among middle-aged and older people. This suggests that targeted interventions for middle-aged and older individuals with sarcopenic obesity are needed in public health efforts to reduce the incidence of hypertension, thereby lowering the risks of complications such as cardiovascular diseases, stroke, and kidney diseases. However, there are some limitations to our study. Firstly, although the formula for ASM has been validated in a Chinese population and has shown good agreement with DXA in various studies, muscle mass was estimated using the formula, not DXA or bioimpedance analysis. This is because there is no DXA or BIA data in CHARLS. This formula has been reported to be in good agreement with DXA and has been used in many studies on sarcopenia, e.g., in Chen et al. (43), Luo et al. (44), Gao et al. (45), Zhou et al. (46) to assess muscle mass. Secondly, the diagnosis of hypertension was based on a questionnaire, not on the results of multiple measurements of blood pressure on different dates, certain hypertension susceptibility factors such as genetic susceptibility, sodium intake, physical activity, environmental and psychological factors were not considered. Thirdly, BMI does not distinguish between fat and muscle mass, which may affect its accuracy in identifying sarcopenic obesity.

## 5 Conclusion

There is no significant correlation between sarcopenia and hypertension, but obesity and sarcopenic obesity increase the risk of hypertension. Targeted management of middle-aged and older people with sarcopenic obesity is needed in public health efforts.

## Data Availability

The datasets presented in this study can be found in online repositories. The names of the repository/repositories and accession number(s) can be found at: https://charls.pku.edu.cn/.
